# Supplementary material for: FSP1 and histone deacetylases suppress cancer persister cell ferroptosis
Source: bioRxiv. 2025 Aug 25:2025.08.21.671520. Preprint. [Version 1] doi: 10.1101/2025.08.21.671520 (PMC12407746; doi:10.1101/2025.08.21.671520)
Supplement: Supplement 1 [file media-1.pdf]

Supplementary Materials for

Cancer persister cell ferroptosis sensitivity is driven by oxidative stress

Masayoshi Higuchi *et al.*

*Corresponding author. Email: mhangauer@health.ucsd.edu

This PDF file includes:

Figs. S1 to S6
Uncropped western blot images
Captions for Supplementary tables S1-S8

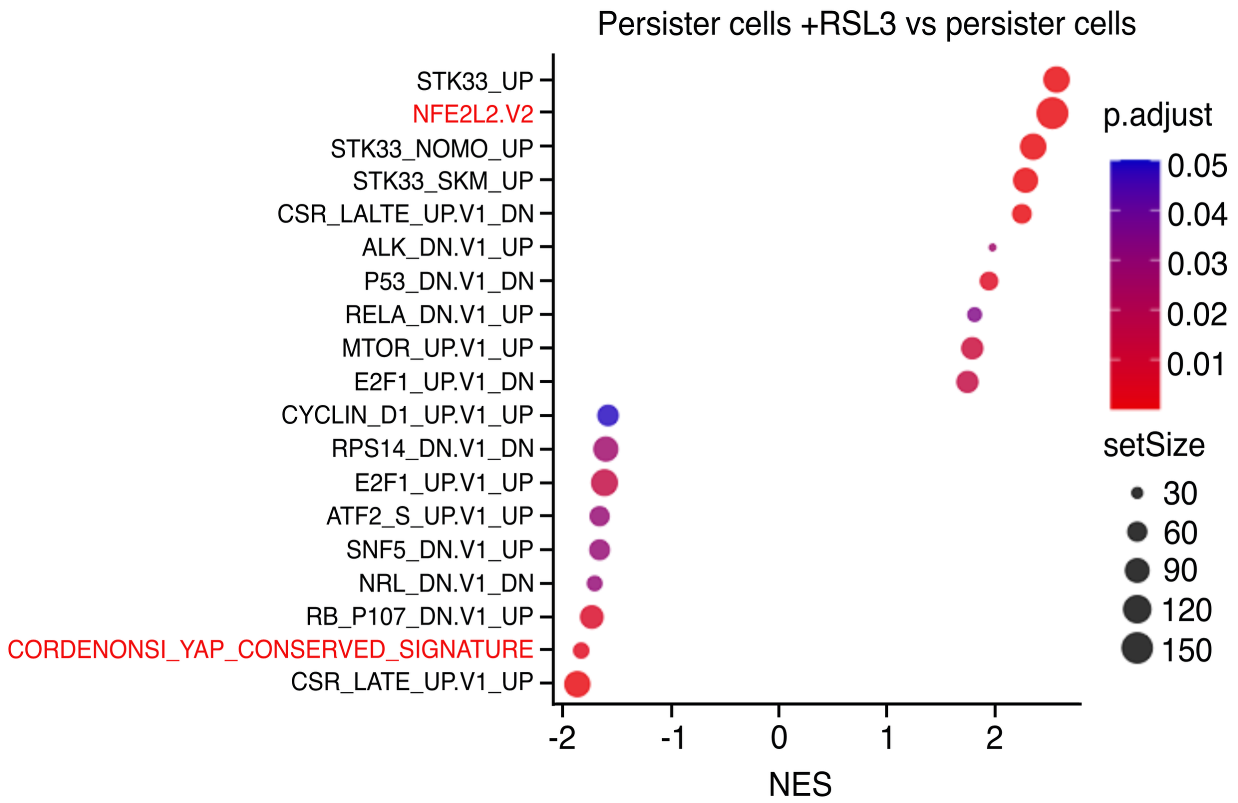

Fig. S1. Enriched Oncogenic Signature gene sets between persister cells treated with or without RSL3. Positive Normalized Enrichment Score (NES) values indicate increased gene set expression in cells treated with RSL3. NFE2L2.V2 refers to the NRF2 gene set.

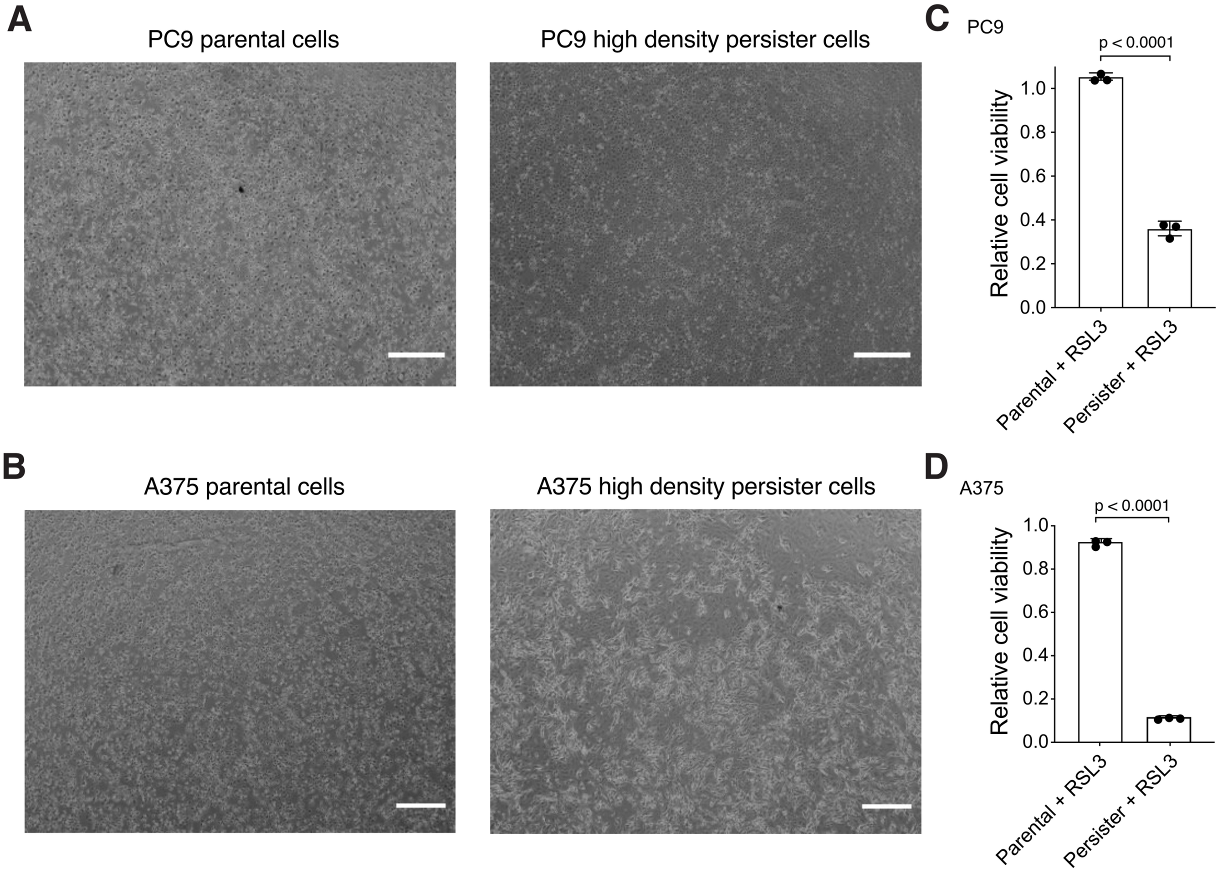

Fig. S2. Persister cells remain sensitized to ferroptosis at high confluency. (**A** and **B**) High density persister cells derived from 70 nM erlotinib for PC9 cells and 10 nM dabrafenib and 1 nM trametinib for A375 cells. Parental cells of similar density are also shown. See Materials and Methods. (**C** and **D**) Cell viability of PC9 and A375 high density persister cells and density-matched parental cells treated with 1 μM RSL3 for 24 hours. n = 3 biological replicates; mean ± s.d. is shown; P values calculated with two-tailed Student's t-test. Scale bar is 100 μm.

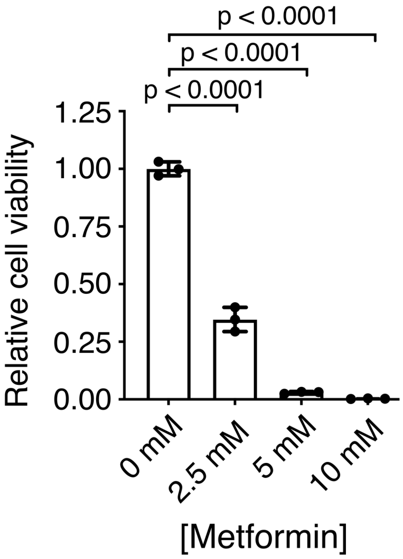

Fig. S3. Metformin is toxic to persister cells. PC9 persister cells derived from treatment with 70 nM erlotinib for 10 days were cotreated with the indicated metformin concentrations throughout drug treatment. $n = 3$ biological replicates; mean ± s.d. is shown; P values calculated with two-tailed Student's t-test.

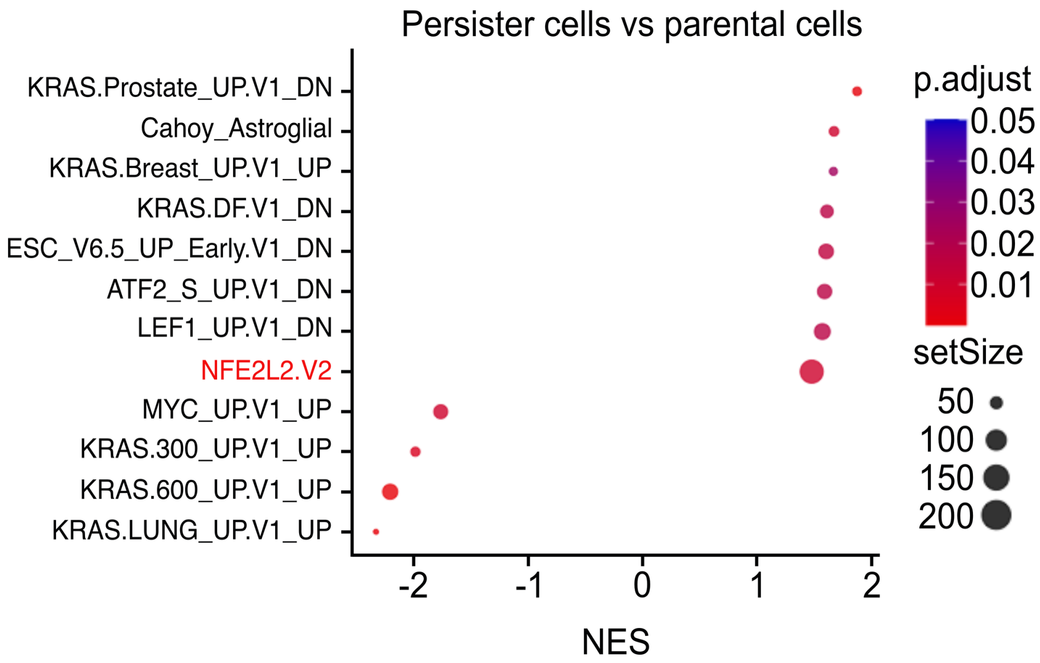

Fig. S4. Enriched Oncogenic Signatures gene sets between PC9 parental and persister cells. Positive Normalized Enrichment Score (NES) values indicate gene sets upregulated in persister cells. NFE2L2.V2 refers to the NRF2 gene set.

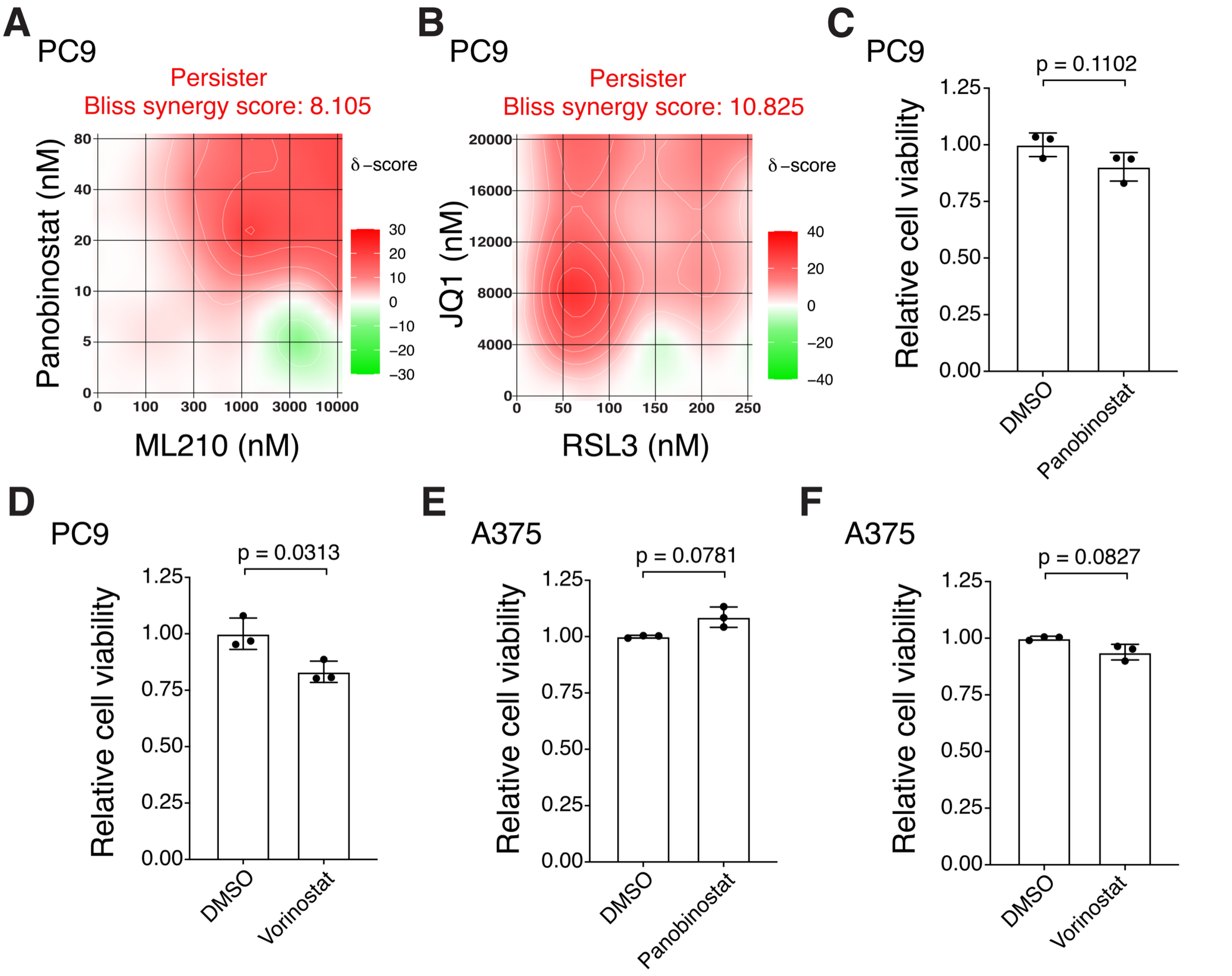

Fig. S5. Additional data demonstrating HDAC and BRD4 inhibitors synergize with GPX4 inhibitors to kill persister cells. (**A** and **B**) Heatmaps of synergy between GPX4 inhibitors ML210 (**A**) or RSL3 (**B**) and HDAC inhibitor panobinostat (**A**) or BRD4 inhibitor JQ1 (**B**) following 24-hour cotreatment of pre-derived PC9 persister cells. Bliss synergy score calculated with SynergyFinder 3.0. (**C** to **F**) Persister cells are insensitive to HDAC inhibitor concentrations which sensitize persister cells to ferroptosis. PC9 and A375 persister cell viability was measured following 48 hour treatment with 7.5 nM panobinostat (**C**), 100 nM vorinostat (**D**), 5 nM panobinostat (**E**), or 1 μM vorinostat (**F**). (**C-F**) n = 3 biological replicates; mean ± s.d. is shown; P values calculated with two-tailed Student's t-test.

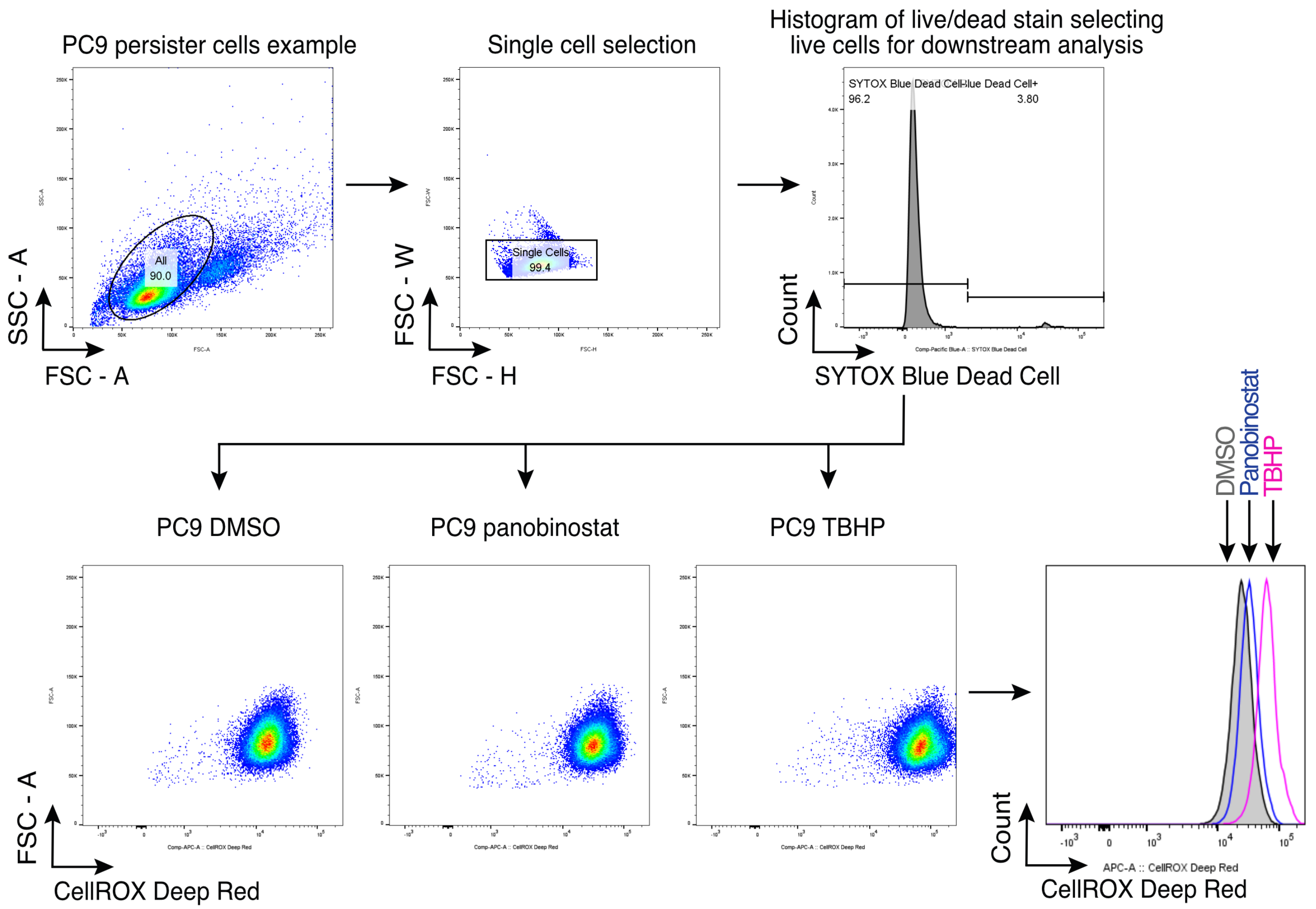

Fig. S6. Flow cytometry schematic for CellROX Deep Red oxidative stress assay. Gating strategy used for Fig. 4G, where geometric means of CellROX deep red signal from live, single cells are graphed. PC9 persister cells were co-treated with DMSO or 7.5 nM panobinostat for 48 hours. Positive control cells were treated with 200 µM tert-butyl hydroperoxide (TBHP) for 1 hour.

Uncropped western blot images

For uncropped western blot images, red boxes indicate bands shown in the main figure while blue boxes indicate loading controls that were not included in the main figures.

Fig. 2A

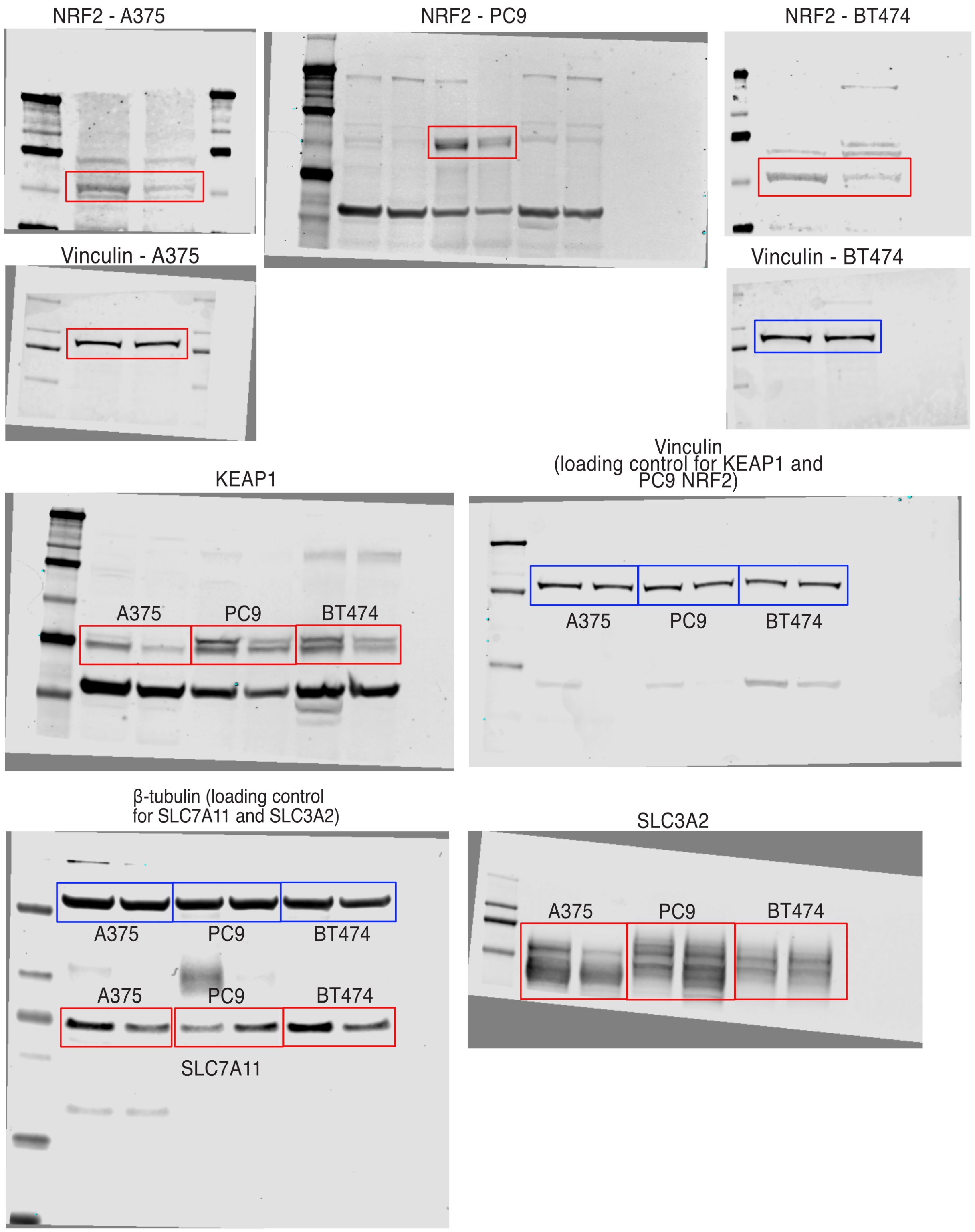

Fig. 2D

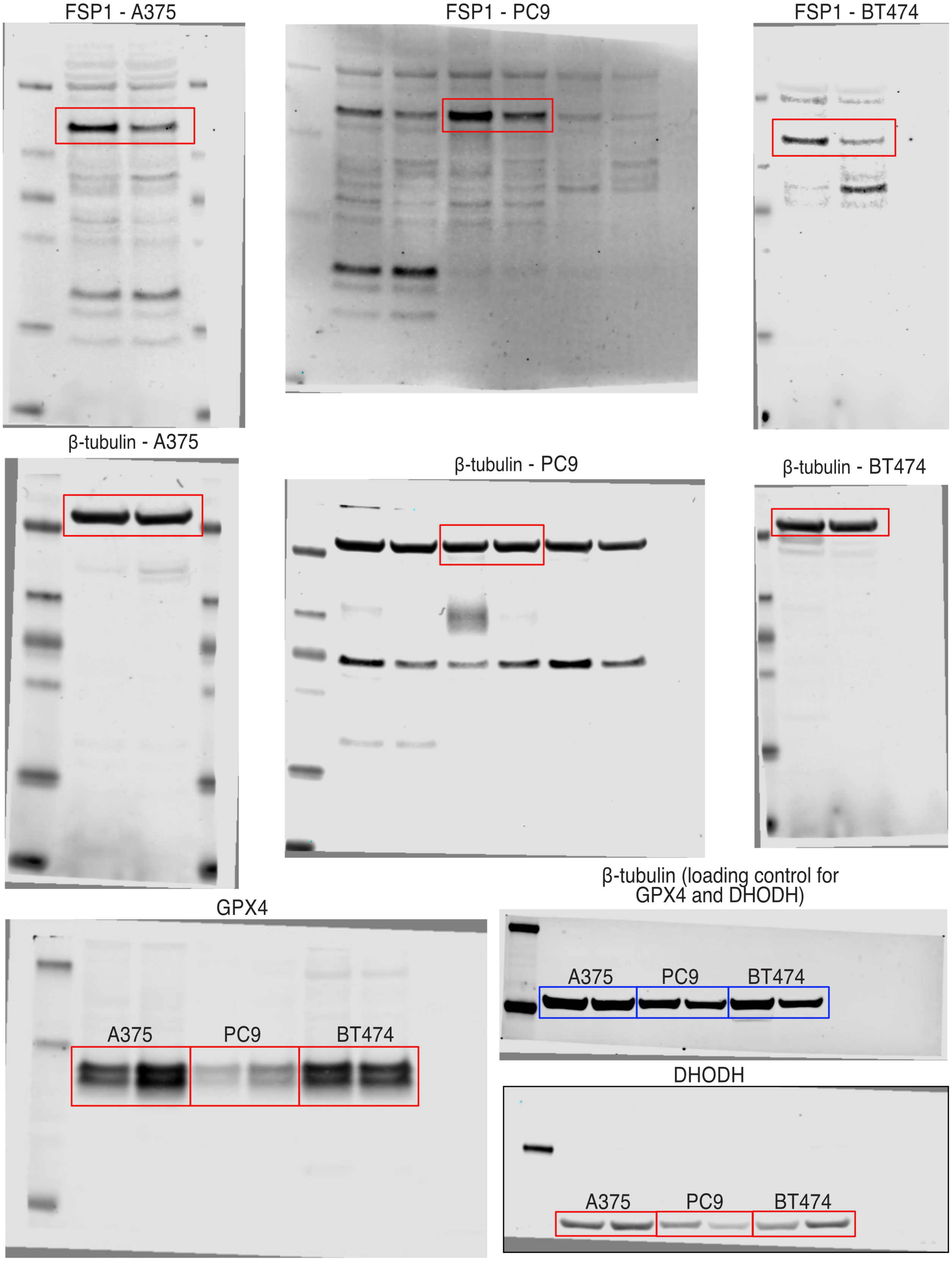
FSP1 - A375
FSP1 - PC9
FSP1 - BT474
β-tubulin - A375
β-tubulin - PC9
β-tubulin - BT474
GPX4
A375
PC9
BT474
β-tubulin (loading control for
GPX4 and DHODH)
A375
PC9
BT474
DHODH
A375
PC9
BT474

Fig. 2D (continued)

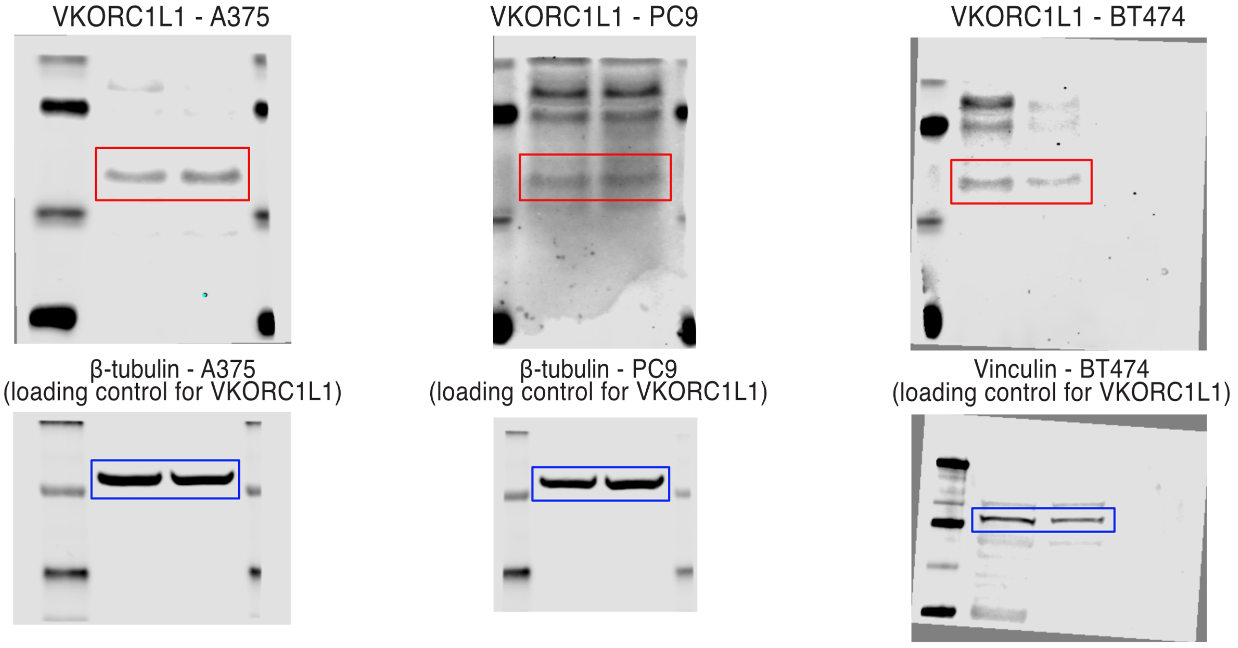

Captions for Tables S1-S8

Table S1. Differentially expressed genes between PC9 persister cells treated with RSL3 versus without RSL3.

Table S2. Enriched Hallmarks and Oncogenic Signatures gene sets between PC9 persister cells treated with RSL3 versus without RSL3.

Table S3. Differentially expressed genes between PC9 persister cell clusters treated with RSL3 versus without RSL3.

Table S4. Enriched Hallmarks and Oncogenic Signatures gene sets between PC9 persister cell clusters treated with RSL3 versus without RSL3.

Table S5. Differentially expressed genes between PC9 persister and parental cells.

Table S6. Enriched Hallmarks and Oncogenic Signatures gene sets between PC9 persister and parental cells.

Table S7. Differentially expressed genes between PC9 persister cells treated with panobinostat versus without panobinostat.

Table S8. Enriched Hallmarks gene sets between PC9 persister cells treated with panobinostat versus without panobinostat.
